# The Cause of Beri-Beri

**Published:** 1905-11-04

**Authors:** 


### The Cause of Beri-beri.

The *Times* paper of last Saturday contains a communicated article, headed "from a Correspondent," which claims for Dr. Hose, lately of Sarawak, that he has succeeded in tracing the causation of beri-beri to the consumption of rice which has become "mouldy," or which, in other words, has been invaded by some still unidentified and undescribed fungus to which poisonous properties are attributed. Dr. Hose furnished a paper to the same effect to the August meeting of the British Medical Association; and the communication to the *Times* appears to belong to the order of those premature disclosures of medical conjectures of which the world has seen only too many examples of late years. For the sake of humanity we cannot but hope that Dr. Hose's conclusions will stand the tests to which they must be subjected; but, until they have emerged from these triumphantly, they can only be regarded as subjects for suspended judgment. We are told that Professor Sims Woodhead is engaged in conducting experiments upon the subject, and that his results will be made public as soon as any satisfactory conclusion can be founded upon them.

According to the article to which we refer, Dr. Hose was led to his presumed discovery by observing that, in Borneo, beri-beri is a disease more prevalent among males than among females, with the result that he endeavoured to find, in the habits of the two sexes, some circumstance which might explain this difference of liability. He observed that the men were accustomed to spend long periods in the jungles, hunting or collecting rubber, and that they took with them (on starting) a stock of food consisting of rice already husked and fit for cooking; while the women, who stayed at home, kept their rice in its husks and prepared it day by day as it was wanted. Further investigation seemed to show that the husk afforded a considerable protection to the enclosed grain, which, when it was stripped and exposed to damp, was very liable to become "mouldy"—that is to say, to be attacked by the fungus in question. Dr. Hose appears to have been medical officer to a prison in Sarawak in which beri-beri was not uncommon among the prisoners, and his first suspicions as to the causation of the disease were confirmed by the results of taking especial care as to the quality of the rice that was supplied to them. As a consequence of this care—or, at least, so it is assumed—the healthy prisoners no longer contracted beri-beri, and those who were already suffering recovered. Since his return to England Dr. Hose has examined many samples of rice, and has discovered not only that much inferior rice is to be met with in the English market, but also that this inferior rice is used by brewers—a circumstance which suggests to him another possible source of the so-called "alcoholic" neuritis from beer drinking. The suggestion is interesting, because the neuritis which prevailed epidemically in the north of England as a consequence of the contamination of beer with arsenic led many persons to suppose that the adjective "alcoholic" was never appropriate, and that all the forms of neuritis to which it had been applied were possibly, or even probably, susceptible of some other explanation. Only two things appear to be even approximately certain in the present stage of the inquiry into the cause of beri-beri, one being that the estimation of the effect of any particular kind of food in relation to a prevalent disease is a matter of extreme difficulty, requiring for its correct performance a very high degree of scientific caution; and the other being that previous inquirers have thought themselves able to exclude diet from among even the probable causes of beri-beri. If the recoveries in the prison described by Dr. Hose are substantiated by more extended experience, it ought not to be quite impossible to bring the question to the test of direct experiment even upon the human subject, for a disease which, even in its slighter forms, answers to that final test of causation which is expressed by the words "*sublata, tollit,*" is surely one which might be permitted to arise without serious scruple. We should have been more hopeful of Dr. Hose's alleged discovery if more caution had been displayed in its presentment; but we are none the less sincere in the hope that the expectations aroused by his work may be fulfilled, and that we may have to congratulate him upon a signal victory against one of the scourges of the tropics.

